# Arginine 199 and Leucine 208 Have Key Roles in the Control of Adenosine A_2A_ Receptor Signalling Function

**DOI:** 10.1371/journal.pone.0089613

**Published:** 2014-03-03

**Authors:** Nicolas Bertheleme, Annette Strege, Sorrel E. Bunting, Simon J. Dowell, Bernadette Byrne

**Affiliations:** 1 Department of Life Sciences, Imperial College London, London, United Kingdom; 2 Department of Molecular Discovery Research, GlaxoSmithKline, Hertfordshire, United Kingdom; University of Cambridge, United Kingdom

## Abstract

One successful approach to obtaining high-resolution crystal structures of G-protein coupled receptors is the introduction of thermostabilising mutations within the receptor. This technique allows the generation of receptor constructs stabilised into different conformations suitable for structural studies. Previously, we functionally characterised a number of mutants of the adenosine A_2A_ receptor, thermostabilised either in an agonist or antagonist conformation, using a yeast cell growth assay and demonstrated that there is a correlation between thermostability and loss of constitutive activity. Here we report the functional characterisation of 30 mutants intermediate between the Rag23 (agonist conformation mutant) and the wild-type receptor using the same yeast signalling assay with the aim of gaining greater insight into the role individual amino acids have in receptor function. The data showed that R199 and L208 have important roles in receptor function; substituting either of these residues for alanine abolishes constitutive activity. In addition, the R199A mutation markedly reduces receptor potency while L208A reduces receptor efficacy. A184L and L272A mutations also reduce constitutive activity and potency although to a lesser extent than the R199A and L208A. In contrast, the F79A mutation increases constitutive activity, potency and efficacy of the receptor. These findings shed new light on the role individual residues have on stability of the receptor and also provide some clues as to the regions of the protein responsible for constitutive activity. Furthermore, the available adenosine A_2A_ receptor structures have allowed us to put our findings into a structural context.

## Introduction

Previous research has shown that mutating a number of residues can both thermostabilise G-protein coupled receptors and modify receptor pharmacology [1]–[4]. Using alanine scanning mutagenesis coupled with ligand binding analysis, it has been possible to generate mutants that are stabilised in different conformations. This approach has led to a number of different high resolution structures of the turkey β_1_ adrenergic receptor [5], [6], the human adenosine A_2A_ (A_2A_R) receptor [7], [8], the neurotensin receptor [9] and the corticotropin-releasing factor receptor 1 [10].

In the case of the A_2A_R, a thermostabilised construct, A_2A_-StaR2, modified to include eight thermostabilising mutations, a further point mutation to remove an N-linked glycosylation site and a C-terminal truncation yielded three different high-resolution crystal structures of the inactive state receptor. Further mutagenesis in the presence of 5′-*N*-Ethylcarboxamidoadenosine (NECA) resulted in an alternative construct, GL31 containing L48A, A54L, T65A, Q89A and N154A substitutions, in a preferentially agonist-bound conformation. This construct yielded structures of the receptor in complex with both the agonist NECA (PDB accession code: 2YDV) and the natural ligand adenosine (PDB accession code: 2YDO). The conformation of the receptor in these structures is suggested to be intermediate between the active and inactive states [8]. Thus, this mutagenesis approach has the potential to facilitate the determination of high resolution GPCR structures in a number of different conformations.

Recent work in our group has explored the effect that thermostabilisation by mutagenesis has on receptor signalling activity [11]. Using a yeast functional assay which allows distinction between agonist-induced and constitutive receptor activities, we were able to show a correlation between the increased thermostability of the Rant5, Rant21 and Rag23 mutants [2] of the A_2A_R and the loss of constitutive activity.

Analysis of the mutants intermediate between the Rant5, which is in an inactive conformation, and the wild-type (WT) revealed that the effects of the mutations on the pharmacological profile of the receptor were additive [11]. The results of this analysis confirmed earlier findings [12] showing the importance of Threonine 88 for agonist binding and activation, although our study also revealed that the T88A mutant retained the ability, albeit markedly reduced, to bind to the natural ligand adenosine [11]. Thus the thermostable mutants generated for structural studies together with the yeast functional assay provide a means of gaining greater insight into the roles of individual amino acids in the mechanism of action of the receptor.

Here we have explored the mutants intermediate between the WT A_2A_R and Rag23 [2], a thermostabilised mutant in an agonist binding conformation containing the following five mutations: F79A, A184L, R199A, L208A and L272A. All possible combinations of the mutations were generated, leading to a total of 30 constructs, which were characterised in a yeast signalling assay [13]. The data showed that R199 and L208 have important roles in receptor function; substituting either of these residues for alanine abolishes constitutive activity. In addition the R199A mutant markedly reduces receptor potency while L208A reduces receptor efficacy. These findings shed new light on the role individual residues have in stability of the receptor and also provide some clues as to the regions of the protein responsible for constitutive activity. Furthermore, the available adenosine A_2A_ receptor structures have allowed us to put our findings into a structural context.

## Materials and Methods

### Material

Yeast nitrogen base (YNB) and yeast extract were purchased from Difco. Peptone, amino acids and 3-Amino Triazole (3AT) were obtained from Sigma-Aldrich and Dimethyl Sulphoxide (DMSO) from Acros Organics. NECA was obtained from Tocris. The Lightning Quikchange site directed mutagenesis kit was obtained from Stratagene/Agilent. Fluorescein-Di-β-D-glucopyranoside was purchased from Invitrogen.

### Construct generation and mutagenesis

The Rag23 A_2A_R mutant was obtained from GeneArt (Regensburg, Germany). The synthetic gene encoded the full-length A_2A_R gene and contained a FLAG tag at the N terminus. The gene was cloned into the pDDGFP *S. cerevisiae* expression plasmid [14]. The construct comprises the receptor gene upstream of the gene coding for GFP-His8. The pDDGFP plasmid was then digested using BamHI and HindIII which excised the complete gene coding for the A_2A_R + GFP-His8 fusion protein. This gene was then ligated into the integrating p306GPD [13] vector. The intermediate mutants were generated from the Rag23 synthetic gene by site-directed mutagenesis using the QuickChange Lightning Site-Directed Mutagenesis kit and the primers detailed in Table S1 in File S1. A complete list of all the mutants can be found in Table 1.

**Table 1:** Characteristics of the A_2A_R constructs used in this study.

	F79A	A184L	R199A	L208A	L272A	Constitutive Activity (% of maximal activity of WT)	Potency (pEC_50_)	Efficacy (% of maximal activity of WT)
**Rag23**	x	x	x	x	x	9%*	7.9±0.2	66±2%
**Rag 23.1**	x	x	x	-	x	4±2%	7.1±0.2	88±7%
**Rag 23.2**	x	-	x	x	x	1±1%	6.6±0.1	30±3%
**Rag 23.3**	-	x	x	x	x	0%*	6.1±0.1	43±1%
**Rag 23.4**	x	x	x	x	-	3±1%	6.9±0.1	33±2%
**Rag 23.5**	x	x	-	x	x	3%*	6.8±0.1	46±3%
**Rag 23.6**	-	-	x	x	x	0%*	6.6±0.1	45±1%
**Rag 23.7**	-	x	-	x	x	1%*	6.7±0.1	53±1%
**Rag 23.8**	-	x	x	-	x	2±2%	6.7±0.3	53±5%
**Rag 23.9**	-	x	x	x	-	0%*	6.4±0.2	59±7%
**Rag 23.10**	x	-	-	x	x	3±2%	6.9*	26±3%
**Rag 23.11**	x	-	x	-	x	2±1%	7.0±0.2	79±4%
**Rag 23.12**	x	-	x	x	-	3%*	6.8±0.2	55±12%
**Rag 23.13**	x	x	-	-	x	71%*	9.8±1.6	86±4%
**Rag 23.14**	x	x	-	x	-	36±6%	7.2±0.2	69±7%
**Rag 23.15**	x	x	x	-	-	1%*	6.5±0.1	67±3%
**Rag 23.16**	-	-	-	x	x	1%*	6.3±0.3	58±7%
**Rag 23.17**	-	-	x	-	x	1%*	5.9±0.1	98±3%
**Rag 23.18**	-	-	x	x	-	0%*	6.1±0.2	60±10%
**Rag 23.19**	-	x	-	-	x	2±1%	6.7±0.1	88±5%
**Rag 23.20**	-	x	-	x	-	4±3%	7.2±0.2	47±4%
**Rag 23.21**	-	x	x	-	-	1±1%	6.6±0.1	85±6%
**Rag 23.22**	x	-	-	-	x	34±2%	7.2±0.1	91±1%
**Rag 23.23**	x	-	-	x	-	8±1%	7.4±0.2	82±3%
**Rag 23.24**	x	-	x	-	-	14±2%	7.4±0.1	85±1%
**Rag 23.25**	x	x	-	-	-	72±10%	8.0±0.4	123±1%
**Rag 23.26**	-	-	-	-	x	15±1%	6.4±0.1	95±5%
**Rag 23.27**	-	-	-	x	-	1±1%	6.4±0.1	58±4%
**Rag 23.28**	-	-	x	-	-	0%*	6.4±0.1	93±5%
**Rag 23.29**	-	x	-	-	-	0%*	6.3±0.1	63±4%
**Rag 23.30**	x	-	-	-	-	76±4%	7.9±0.4	95±1%
**WT**	-	-	-	-	-	44±1%	7.1±0.3	94±1%

For each of the constructs shown an “x” indicates the presence of a specific mutation while a “-” indicates the presence of the WT amino acid. The constitutive activity, potency and efficacy values for each receptor are shown. Each parameter is an average of two independent experiments performed in triplicate.

*No errors are given in these examples as the data obtained from different experiments was so consistent as to preclude their calculation.

### Expression

All the A_2A_R constructs were fusions with a C-terminal GFP-8His tag in the p306GPD vector, transformed using the lithium-acetate procedure [15] and chromosomally integrated at the *ura3* locus in the MMY24 (*MAT*a *fus1*::*FUS1-HIS3 LEU2*::*FUS1-lacZ far1 sst2 ste2 gpa1*::*ADE2 his3 ura3 TRP1::GPA1-Gαi3*) yeast strain [13]. Estimation of the expression levels for all the constructs using GFP as previously described [14] gave values between 0.4–1.4 mg/ml (Table S2 in File S1).

### Yeast cell growth assay

The yeast cell growth assay was performed as previously described [11]. Briefly, the individual colonies of MMY24 cells containing each mutant were inoculated into Synthetic Complete medium lacking uracil (‘–URA medium’; 6.7% YNB, 2% D-glucose, 1.26 g/L amino acid supplement (23.53 mg of L-arginine (HCl), 117.6 mg of L-aspartic acid, 117.6 mg of glutamic acid (monosodium), 35.29 mg of L-lysine, 23.53 mg of L-methionine, 58.82 mg of L-phenylalanine, 441.2 mg of L-serine, 235.3 mg of L-threonine, 35.29 mg of L-tyrosine and 176.5 mg of L-valine) supplemented with histidine to a final concentration of 20 mg/L, for 17 hours at 30°C. The cultures obtained were diluted into –URA medium supplemented with 26.1 mM Na_2_HPO_4_.7H_2_O, 21.1 mM NaH_2_PO_4_, pH 7.0 to an OD_600_ of 0.02. The assay mix was supplemented with 3AT, to a final concentration of 5 mM. FDGlu, a substrate of exoglucanase, an endogenous yeast enzyme secreted from dividing cells, was also added to the medium to a final concentration of 20 µM. The product of this reaction is the fluorescent molecule, fluorescein. An increase in fluorescence (excitation wavelength = 485 nm, emission wavelength = 535 nm) is thus a measure of growth of the culture, which is a measure of receptor activity. Different concentrations of agonist (0.17 pM-0.2 mM), were added. The yeast growth was assessed by fluorescence measurement using a Spectramax M2e platereader (Molecular Devices) following 23 h incubation at 30°C. Log_10_ [NECA] against fluorescence curves were plotted and fitted to non-linear regression, providing EC_50_ values. Data were analyzed using GraphPad Prism 6.0 (GraphPad Software, San Diego, CA, USA). All the data were normalized to WT.

## Results and Discussion

### Functional profiles of the Wild-Type and Rag23 receptors

**Figure 1:**
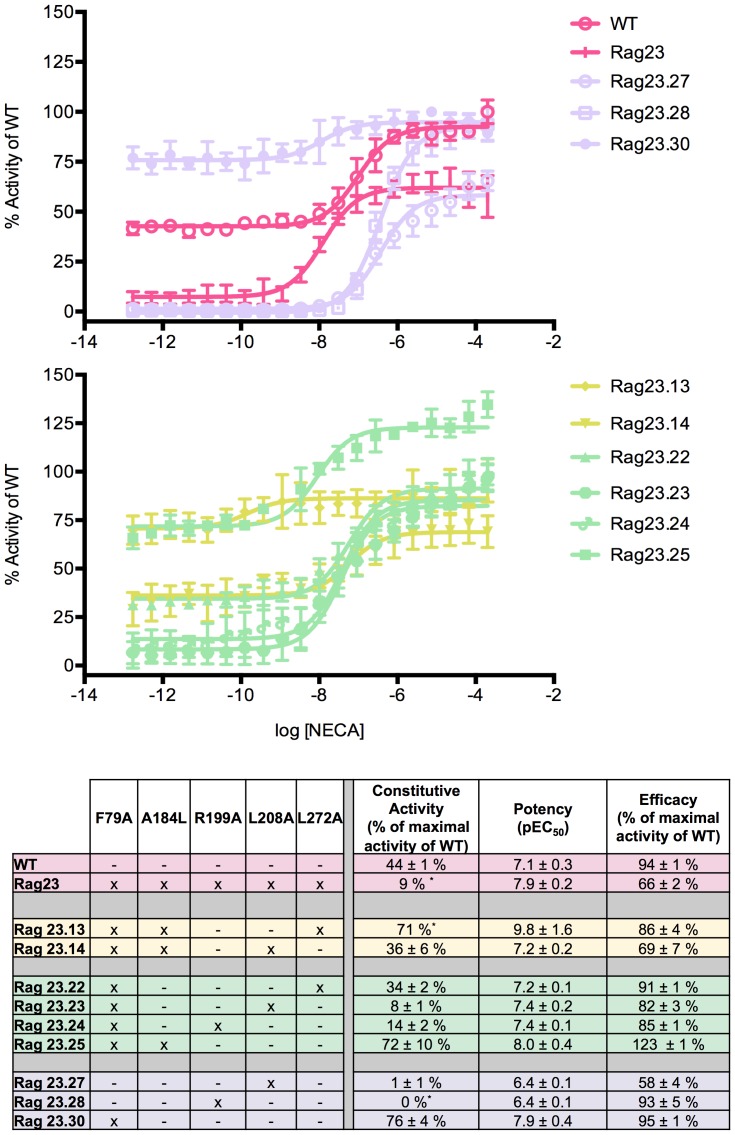
Functional profiles of constructs illustrating the major effects of the F79A mutation. The F79A mutation increases constitutive activity, efficacy and potency, both alone (Rag23.30) and in combination with other mutations (Rag23.13, Rag23.14, Rag23.22, Rag23.23, Rag23.24, Rag23.25, Rag23.27, Rag23.28 and Rag23.30). The curves (A and B) are the average of two experiments performed in triplicate. The table (C) shows the pharmacological profile of each mutant characterized. The colours of the curves correspond to those in the table. The data for the WT and Rag23 constructs (pink) are also shown for comparison.

**Figure 2:**
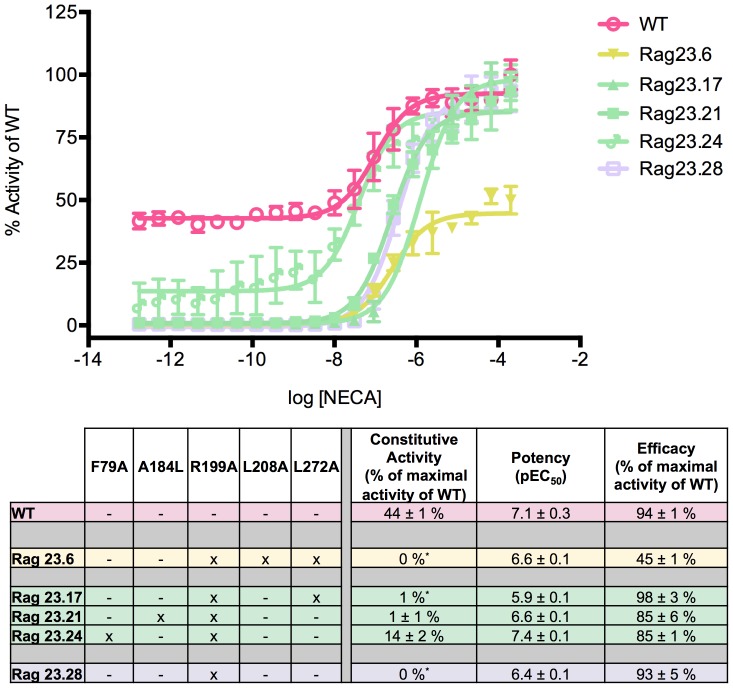
Functional profiles of constructs illustrating the major effects of the R199A mutation. The R199A mutation reduces constitutive activity and potency, both alone (Rag23.28) and in combination with other mutations (Rag23.6, Rag23.17, Rag23.21, Rag23.24 and Rag23.28). The curves (A) are the average of two experiments performed in triplicate. The table (B) shows the pharmacological profile of each mutant characterized. The colours of the curves correspond to those in the table. The data for the WT (pink) are also shown for comparison.

**Figure 3:**
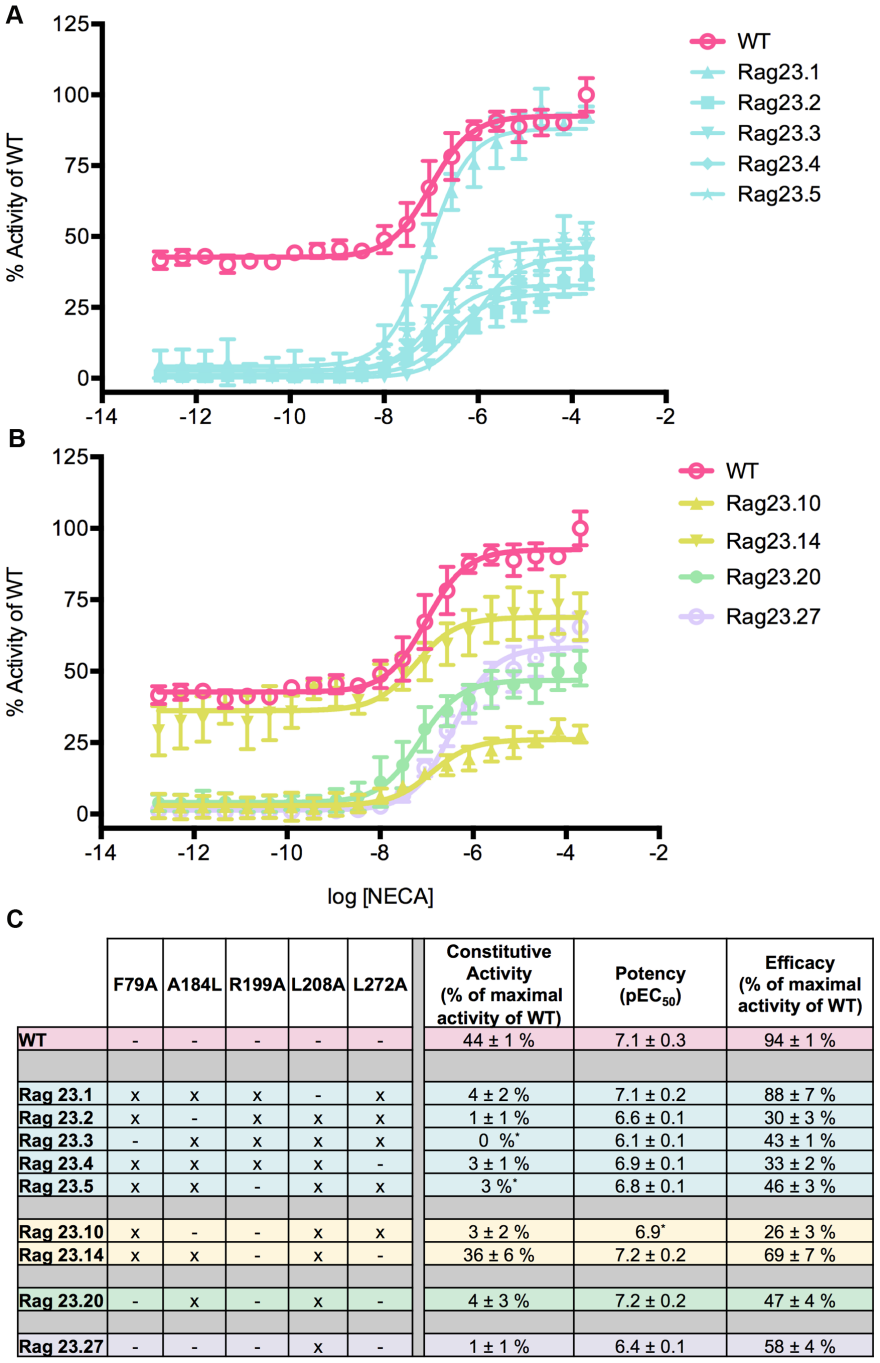
Functional profiles of constructs illustrating the major effects of the L208A mutation. The L208A mutation reduces constitutive activity and efficacy, both alone (Rag23.27) and in combination with other mutations (Rag23.1, Rag23.2, Rag23.3, Rag23.4, Rag23.5, Rag23.10, Rag23.14, Rag23.20 and Rag23.27). The curves are the average of two experiments performed in triplicate. The table (C) shows the pharmacological profile of each mutant characterized. The colours of the curves correspond to those in the table. The data for the WT (pink) are also shown for comparison.

**Figure 4:**
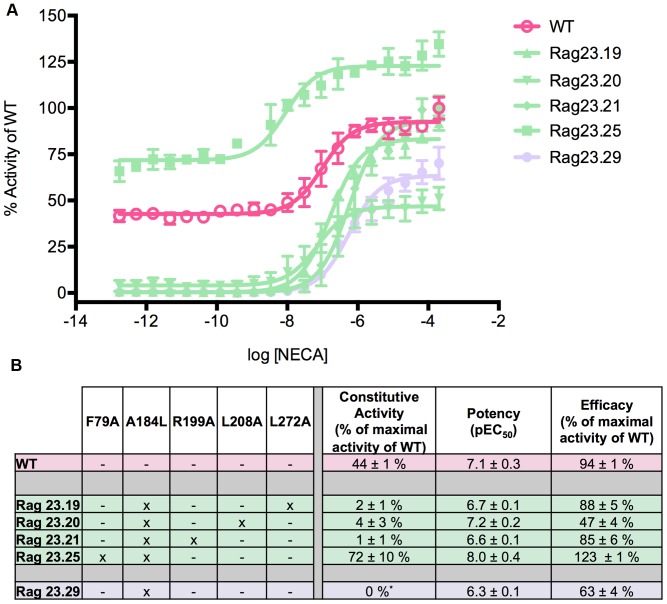
Functional profiles of constructs illustrating the major effects of the A184L mutation. The A184L mutation alone abolishes constitutive activity (Rag23.29) but this is overcome by the dominant effect of F79A (Rag23.25). The curves (A) are the average of two experiments performed in triplicate. The table (B) shows the pharmacological profile of each mutant characterized. The colours of the curves correspond to those in the table. The data for the WT (pink) are also shown for comparison.

**Figure 5:**
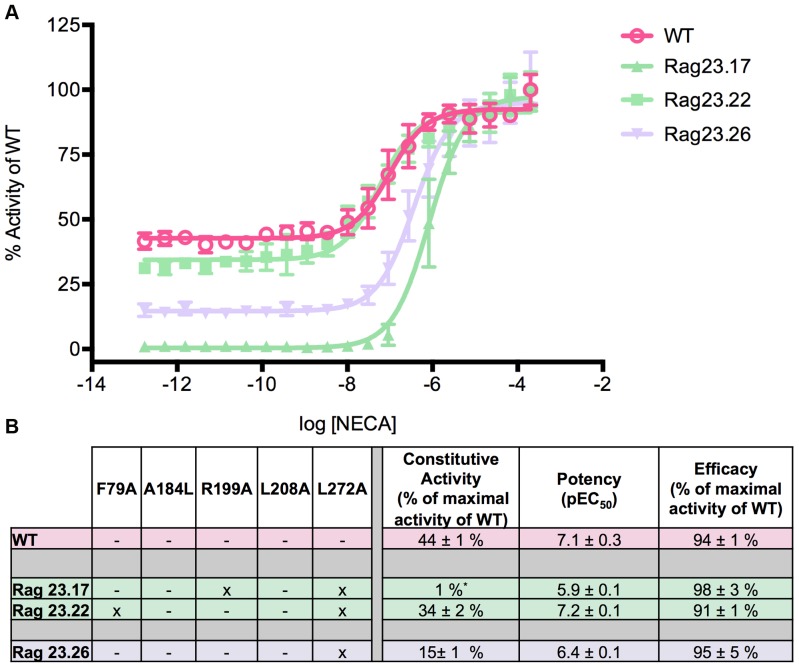
Functional profiles of constructs illustrating the major effects of the L272A mutation. The L272A mutation alone decreases constitutive activity on its own (Rag23.26) but this effect is overcome by the dominant effect of the F79A (Rag23.22). In the case of the combination with the R199A (Rag23.17) mutation there is an almost complete loss of constitutive activity. The curves (A) are the average of two experiments performed in triplicate. The table (B) shows the pharmacological profile of each mutant characterized. The colours of the curves correspond to those in the table. The data for the WT (pink) are also shown for comparison.

The activity of each of the receptor constructs is summarised in Table 1. The numerical data for some of the mutants is also shown in Figures 1–5C. The graphical data for all constructs is shown in S1–4 in File S1. The WT (indicated in pink in Figures 1–5) exhibits two distinct activities: constitutive activity in the absence of agonist and agonist induced activity in the presence of increasing concentrations of NECA as observed previously [11]. The constitutive activity makes up almost half of the maximal activity of the WT receptor (Figure 1A, Table 1) in agreement with the high levels of constitutive activity seen for the receptor in mammalian cells [16]. In contrast, the Rag23 construct containing all five of the mutations F79A, A184L, R199A, L208A, L272A exhibits high agonist induced activity but almost no constitutive activity (Figure 1A) as shown previously [11]. In addition Rag23 has an increased potency (pEC_50_) compared to the WT as observed previously [11].

### F79A enhances constitutive activity, potency and efficacy

The effects of the F79A mutation are illustrated by the single mutant Rag23.30. This has increased constitutive activity and potency compared with the WT while retaining similar efficacy (Figure 1A). These effects are also observed in constructs where F79A is combined with A184L and/or L272A (Rag23.13, Rag23.22 and Rag23.25; Figure 1B). All these constructs exhibit WT or higher levels of potency and constitutive activity. In contrast, constructs combining F79A with R199A or L208A (Rag23.23 and Rag23.24; Figure 1B), exhibit markedly lower constitutive activity than the WT. However, the presence of the F79A mutation still increases the constitutive activity of the receptor compared with the equivalent constructs containing R199A or L208A alone (Rag23.27 and Rag23.28; Figure 1A). The same trend is also observed for receptor potency demonstrating that the effects of F79A on potency and constitutive activity are antagonized by the presence of the R199A and L208A. One exception to that is Rag23.14, the combination of F79A, A184L and L208A produces an overall profile similar to WT. It is not clear from either the data presented here or the structures why this WT-like behaviour is seen for this construct.

Interestingly, the F79A mutation is not thermostabilising but was included in the Rag23 because it was preferentially in an agonist-binding conformation [2]. The increased constitutive activity and lack of thermostabilisation of the F79A mutation is consistent with our previous findings demonstrating a correlation between loss of constitutive activity and thermostabilisation [11].

### Structural basis of the effects of the F79A mutation

**Figure 6:**
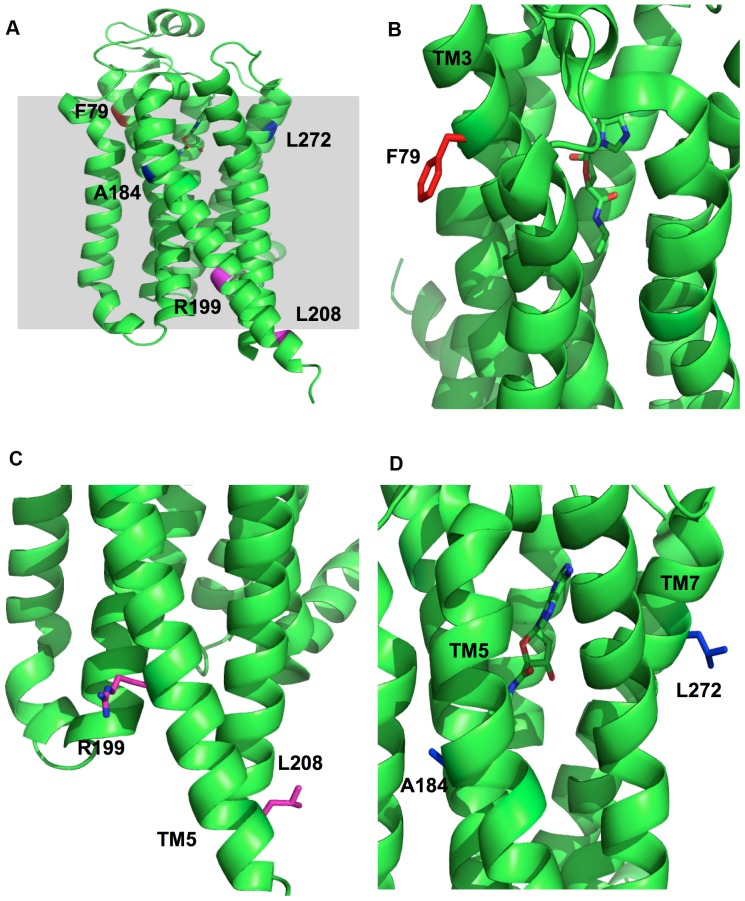
Position of the F79 (red), R199A and L208 (magenta), A184 and L272 (blue) residues in the high-resolution crystal structure of the A_2A_R GL31 thermostable mutant (PDB accession code: 2YDV) (A). F79 is located on TM3 (B) while R199 and L208 are both located at the cytoplasmic end of TM5 (C) and A184 and L272 are located at the extracellular end of TM5 and TM7 respectively (D).

A number of high resolution A_2A_R structures have been obtained using a variety of techniques and in the presence of different ligands [7], [8], [17]–[19]. Given that here we are exploring the roles of mutations in a thermostabilised receptor in the preferentially agonist conformation [2], we have used the structure of a thermostabilised A_2A_R containing 5 point mutations in complex with the agonist NECA [8] to provide further context to the results of this study (PDB accession code: 2YDV). In the structure, F79 is located on trans-membrane helix 3 (TM3; Figure 6A and 6B), a region of the protein with key roles in GPCR structure and function. The recent analysis of the known GPCR structures by Venkatakrishnan et al [20] revealed that TM3 interacts with all other TMs except TMs1 and 7 and is thus central for maintaining the overall GPCR scaffold. Furthermore, residues in TM3 have been shown to form interactions with the ligand in nearly all the receptors for which a high-resolution structure is available [20]. In addition, once the receptor is activated, TM3 forms a critical interaction with the G-protein as exemplified by Arg^3.50^ interacting with a backbone carbonyl of the C terminus of the G protein [21]. It is therefore not a surprise that GPCR activity is very sensitive to mutations in TM3, since these often lead to loss of function or markedly increased constitutive activity [12], [20], [22]. A comparison of the structure of the A_2A_R bound to NECA (PDB accession code: 2YDV) with the structure of the A_2A_R-T4L bound to ZM241385 (PDB accession code: 3EML) reveals a 2 Å upward movement of TM3 in the active-like conformation of the receptor [8]. In the inactive conformation (PDB accession code: 3PWH), F79 forms van der Waals interactions with a number of surrounding residues including F62 and L137. These interactions are lost when F79 is mutated to an alanine possibly resulting in a less stable inactive conformation. The F79A receptor construct is therefore more likely to adopt an active conformation, with the associated 2 Å upward movement of TM3 explaining the increase in constitutive activity observed for this mutant in our study.

### R199A reduces constitutive activity and potency

All constructs containing the R199A mutation exhibited an almost complete lack of constitutive activity and reduced potency compared with the WT (Figure 2). For clarity, Figure 2 only shows these effects for some examples of the intermediate single, double and triple mutants. However the same characteristics are clearly seen in all constructs with the R199A mutation (Table 1). The data strongly indicate that R199 has a key role in the constitutive activity of the A_2A_R. This is further illustrated by Rag23.24, which contains both F79A and R199A mutations. The profile of this mutant shows that the increase of constitutive activity due to the F79A mutation is almost completely overcome by the R199A mutation (Figure 2) and as mentioned above the R199A further reduces the overall constitutive activity of this mutant compared with WT. The R199A mutation also reduces receptor potency compared with WT levels. However, the effects are less dominant than those observed for the constitutive activity. F79A alone increases potency but in combination with R199A produces a construct with WT potency. These findings indicate that the R199A cancels out the increase in potency caused by F79A, but does not further affect potency in this construct compared with WT. The Rag23.6 triple mutant (R199A, L208A and L272) also exhibits no constitutive activity and reduced potency but this construct also shows a markedly reduced efficacy (Figure 2). One possible reason for this could be reduced expression; however, comparison of the expression levels of the different receptors (Table S2 in File S1) shows that Rag23.6 expresses to the same level (1 mg/L) as the WT.

### L208A mutation reduces constitutive activity and efficacy

Almost all the constructs containing the L208A mutations exhibit very low or undetectable constitutive activity and markedly reduced efficacy compared with WT (Figure 3). As for the R199A mutation, the effect of the L208A mutation on the constitutive activity almost completely overcomes the enhancing effect of the F79A, except in the presence of A184L, Rag23.14 (Figure 3B).

The effects of L208A on efficacy are illustrated by the quadruple mutant, Rag23.1 which lacks the L208A substitution and has markedly increased efficacy compared with all the constructs containing L208A. This indicates that the low levels of efficacy observed in Rag23.2, 3, 4 and 5 as well as Rag23 are due to the presence of the L208A mutation (Figure 3A). Although there is variation in the expression level of these mutants, this does not account for the changes in efficacy observed (Table S2 in File S1). Indeed several of these constructs exhibit similar or higher expression levels compared with the WT receptor. The L208A mutation influences the potency in a similar way to the R199A mutation.

### Structural basis of the effects of the R199A and L208A mutations

In the structure of the A_2A_R, R199 and L208 are both located in the cytoplasmic portion of trans-membrane helix 5 (TM5; Figure 6A and 6C). This region of TM5 is involved in the interaction between the β_2_ adrenergic receptor and the G_αs_ [21] and is thus likely to be involved in the interaction of the A_2A_R with the G_α1_-related chimera protein used in the yeast cell growth assay.

Both the R199A and L208A mutations affect the constitutive activity dramatically. However, only the L208A mutation affects the efficacy. The structures of the A_2A_R and the β_2_AR in complex with G_S_ provide clues to explain this difference. Residues R199 and L208 are conserved in the β_2_AR as R221 and L230 respectively. Due to the lack of a structure of the A_2A_R in an active conformation, we used the structure of the β_2_AR in complex with the G_s_ protein to aid interpretation of our data. In this structure, R221 of the β_2_AR forms a hydrogen bond with a threonine (equivalent to an arginine in the A_2A_R) on TM3 when the receptor is in complex with the G_s_ protein. This hydrogen bond no longer exists when R221 is mutated to an alanine. Here we have demonstrated that the R199A mutation completely abolishes constitutive activity of the A_2A_R without affecting efficacy suggesting that the interaction between TM5 and TM3 is crucial for constitutive activity but not for agonist-induced activity.

The L230 residue of the β_2_AR forms a direct interaction with the leucine at the extreme C-terminal end of the G protein. Mutating the equivalent residue on the A_2A_R, L208, seems to prevent constitutive G-protein activation but only reduces agonist induced activation. This suggests that this interaction is crucial for formation of a constitutively active complex but it is of less importance in the formation of the agonist-induced active complex.

The fact that L208 is in direct contact with the G protein, while R199 is involved in making intra-receptor contacts, may explain why the L208A mutation has an effect on both constitutive activity and efficacy while the R199A mutation affects only the constitutive activity.

Our previous study suggested that the agonist induced and constitutively active conformations of the A_2A_R are distinct [11], thus inhibiting constitutive activity of the receptor does not necessarily have any negative effects on agonist-induced activity.

### The effects of A184L and L272A are overcome by the other mutations

As can be seen from the data for the A184L single mutant construct, Rag23.29, this substitution has effects on the three key functional parameters assessed here (Figure 4). It reduces the constitutive activity, potency and efficacy of the receptor (0%, 6.3%, and 63%, respectively; Figure 4B). An almost identical functional profile is seen for Rag23.19 (Figure 4), which combines the A184L and L272A mutations. However, these effects are not observed in those constructs, which combine the A184L with L208A, R199A and/or F79A. For example, when A184L (Rag23.29) is combined with the strong positive effects of F79A on constitutive activity, potency and efficacy (Rag23.30), this leads to an increase in all three parameters of the resulting mutant (Rag23.25; Figure 4) relative to WT. In contrast, addition of A184L with R199A (Rag 23.21; Figure 4) or L208A (Rag23.20; Figure 4) results in an almost complete loss of constitutive activity and reduced potency (with R199A) and efficacy (with L208A).

L272A alone has a moderate negative effect on the constitutive activity and potency of the A_2A_R (Rag23.26, Figure 5). Much like A184L, these effects are markedly influenced by the presence of other more dominant mutations. For example, when L272A is combined with F79A in Rag23.22 (Figure 5), this mutant still has increased constitutive activity and potency compared to WT, as a result of the strong influence of F79A. In contrast, when L272A is combined with R199A in Rag23.17, this mutant has very low levels of constitutive activity and reduced potency compared with WT due to the influence of R199A.

### Structural basis for the effects of the A184L and L272A mutations

A184L and L272A are located on the extracellular ends of TM5 and TM7 respectively (Figure 6A and 6D). Both of these residues are a significant distance from both the ligand-binding pocket and the G protein-binding region. Based on their location in the crystal structure [8] these residues are unlikely to be directly involved in G protein coupling or ligand binding (Figure 6D). The data presented here, together with the available structural data, are not sufficient to explain why A184L and L272A have the observed functional effects in the receptors with these single mutations (i.e. Rag23.29 and Rag23.26). However, this does provide clues as to why F79A, R199A and L208A have more dominant effects on the function of the receptor.

Interestingly, the dominance of the individual mutations is in accordance with their stabilisation effects observed by Magnani and colleagues [2]. One exception is the F79A mutation, which is not thermostabilising [2]. For example, the R199A (Rag23.28) and L208A (Rag23.27) single mutants retain 101% and 108% of the WT binding activity after heating at 30°C for 30 minutes respectively while the A184L (Rag23.29) and L272A (Rag23.26) retained 75% and 79% respectively. In this analysis WT binding activity after heating is taken as 50%. The F79A mutant shows similar levels of activity after heating as the WT receptor.

In conclusion, the R199A and L208A mutations inhibit constitutive activity of the A_2A_R receptor while F79A enhances constitutive activity. In addition, the L208A mutation also affects the efficacy of the receptor. The effects of A184L and L272A are overcome by the more dominant F79A, R199A and L208A mutations. Analysis of the mutations alone and in combination using the yeast assay, together with the known A_2A_R structures provides information on the role of individual amino acids in receptor function. However as with all types of analysis of this kind it can be difficult to fully dissect the activity of an individual amino acid from the contributions of all others. A full understanding of the roles of all the amino acid residues will only be revealed through multiple crystal structures in a range of different conformations coupled with detailed dynamics studies.

## Supporting Information

File S1 **Supporting Figures and Tables. Figure S1, NECA-induced activity of the WT A_2A_R, Rag23 and the quadruple mutants intermediate between the WT and Rag23.** See Table 1 for the precise details of each mutant. The receptor constructs were expressed in the MMY24 *S. cerevisiae* strain using the p306GPD vector. The activity of cells containing empty vector is shown as a control. **Figure S2, NECA-induced activity of the WT A_2A_R and the triple mutants intermediate between the WT and Rag23.** See Table 1 for the precise details of each mutant. The receptor constructs were expressed in the MMY24 *S. cerevisiae* strain using the p306GPD vector. The activity of cells containing empty vector is shown as a control. **Figure S3, NECA-induced activity of the WT A_2A_R and the double mutants intermediate between the WT and Rag23. See** **Table 1** **for the precise details of each mutant.** The receptor constructs were expressed in the MMY24 *S. cerevisiae* strain using the p306GPD vector. The activity of cells containing empty vector is shown as a control. **Figure S4, NECA-induced activity of the WT A_2A_R and the single mutants intermediate between the WT and Rag23.** See Table 1 for the precise details of each mutant. The receptor constructs were expressed in the MMY24 *S. cerevisiae* strain using the p306GPD vector. The activity of cells containing empty vector is shown as a control. **Table S1, Oligos used to generate the mutant receptor constructs. Table S2, Expression levels of the thirty mutants and the wild-type calculated using the eGFP fluorescence as described by Drew** ***et al.*** **(2008, Nature Protocols, 3: 784–798).**
(DOCX)
