# Supplementary material for: Arginine 199 and Leucine 208 Have Key Roles in the Control of Adenosine A2A Receptor Signalling Function
Source: PLoS One. 2014 Mar 3;9(3):e89613. doi: 10.1371/journal.pone.0089613 (PMC3940607; doi:10.1371/journal.pone.0089613)
Supplement: File S1 — Supporting Figures and Tables. Figure S1, NECA-induced activity of the WT A2AR, Rag23 and the quadruple mutants intermediate between the WT and Rag23. See Table 1 for the precise details of each mutant. The receptor constructs were expressed in the MMY24 S. cerevisiae strain using the p306GPD vector. The activity of cells containing empty vector is shown as a control. Figure S2, NECA-induced activity of the WT A2AR and the triple mutants intermediate between the WT and Rag23. See Table 1 for the precise details of each mutant. The receptor constructs were expressed in the MMY24 S. cerevisiae strain using the p306GPD vector. The activity of cells containing empty vector is shown as a control. Figure S3, NECA-induced activity of the WT A2AR and the double mutants intermediate between the WT and Rag23. See Table 1 for the precise details of each mutant. The receptor constructs were expressed in the MMY24 S. cerevisiae strain using the p306GPD vector. The activity of cells containing empty vector is shown as a control. Figure S4, NECA-induced activity of the WT A2AR and the single mutants intermediate between the WT and Rag23. See Table 1 for the precise details of each mutant. The receptor constructs were expressed in the MMY24 S. cerevisiae strain using the p306GPD vector. The activity of cells containing empty vector is shown as a control. Table S1, Oligos used to generate the mutant receptor constructs. Table S2, Expression levels of the thirty mutants and the wild-type calculated using the eGFP fluorescence as described by Drew et al. (2008, Nature Protocols, 3: 784–798). (DOCX) [file pone.0089613.s001.docx]

**Bertheleme et al,**

**Supplementary Information**

**Table S1** Oligos used to generate the mutant receptor constructs

| **Primer** | **Sequence (5' to 3')** |
| --- | --- |
| A79F_forward | gatgttgggtgtttacttgcgtattttcttggctgccaga |
| A79F_reverse | taccaaaacgaagcaagcaataaacaaacaaccatgacaagcagc |
| L184A_forward | catggtctacttcaatttcttcgcgtgtgttttggtccctttgttg |
| L184A_reverse | ggagaagatagaagattgagtcaaaaccaaaacgaagcaag |
| A199R_forward | ctaagtccttggctattatagttggtttgttcgctttgtgttg |
| A199R_reverse | gcaatgatgcccgcagccctcgtgccggtca |
| A208L_forward | ctattttcttggctgccagaagacaactaaagcaaatggaatctcaacc |
| A208L_reverse | ggttgagattccatttgctttagttgtcttctggcagccaagaaaatag |
| A272L_forward | tcatgctccattgtggttgatgtatctagctatagttttgtcccacac |
| A272L_reverse | gtgtgggacaaaactatagctagatacatcaaccacaatggagcatga |

**Table S2** Expression levels of the thirty mutants and the wild-type calculated using the eGFP fluorescence as described by Drew *et al.* (2008, Nature Protocols, 3: 784-798).

|  | **RFU** | **Level of Expression (mg/L)** |
| --- | --- | --- |
| **Rag 23.1** | 16,843 | 0.6 |
| **Rag 23.2** | 20,715 | 1.1 |
| **Rag 23.3** | 22,439 | 1.2 |
| **Rag 23.4** | 19,046 | 0.9 |
| **Rag 23.5** | 16,389 | 0.6 |
|  |  |  |
| **Rag 23.6** | 20,479 | 1.0 |
| **Rag 23.7** | 23,799 | 1.4 |
| **Rag 23.8** | 14,547 | 0.4 |
| **Rag 23.9** | 19,162 | 0.9 |
| **Rag 23.10** | 17,677 | 0.7 |
| **Rag 23.11** | 18,500 | 0.8 |
| **Rag 23.12** | 19,021 | 0.9 |
| **Rag 23.13** | 20,392 | 1.0 |
| **Rag 23.14** | 18,314 | 0.8 |
| **Rag 23.15** | 18,906 | 0.9 |
|  |  |  |
| **Rag 23.16** | 21,423 | 1.2 |
| **Rag 23.17** | 20,992 | 1.1 |
| **Rag 23.18** | 21,782 | 1.2 |
| **Rag 23.19** | 23,018 | 1.3 |
| **Rag 23.20** | 22,388 | 1.2 |
| **Rag 23.21** | 16,312 | 0.6 |
| **Rag 23.22** | 14,803 | 0.4 |
| **Rag 23.23** | 15,874 | 0.5 |
| **Rag 23.24** | 19,569 | 0.9 |
| **Rag 23.25** | 14,356 | 0.4 |
|  |  |  |
| **Rag 23.26** | 15,904 | 0.6 |
| **Rag 23.27** | 16,984 | 0.7 |
| **Rag 23.28** | 19,843 | 1.0 |
| **Rag 23.29** | 20,120 | 1.0 |
| **Rag 23.30** | 18,013 | 0.8 |
|  |  |  |
| **WT** | 19,954 | 1.0 |


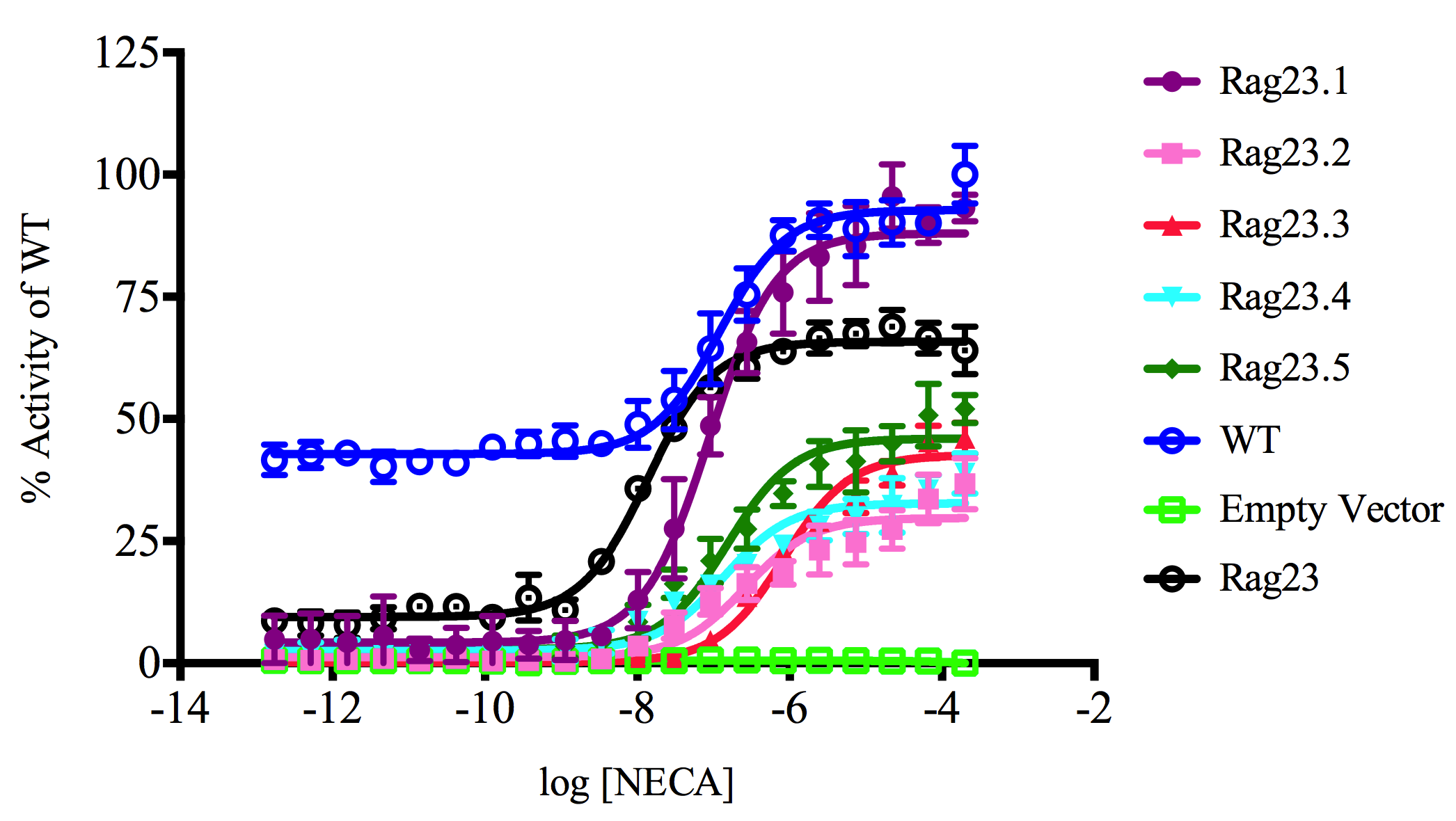


**Figure S1** NECA-induced activity of the WT A_2A_R, Rag23 and the quadruple mutants intermediate between the WT and Rag23. See Table 1 for the precise details of each mutant. The receptor constructs were expressed in the MMY24 *S. cerevisiae* strain using the p306GPD vector. The activity of cells containing empty vector is shown as a control.

**
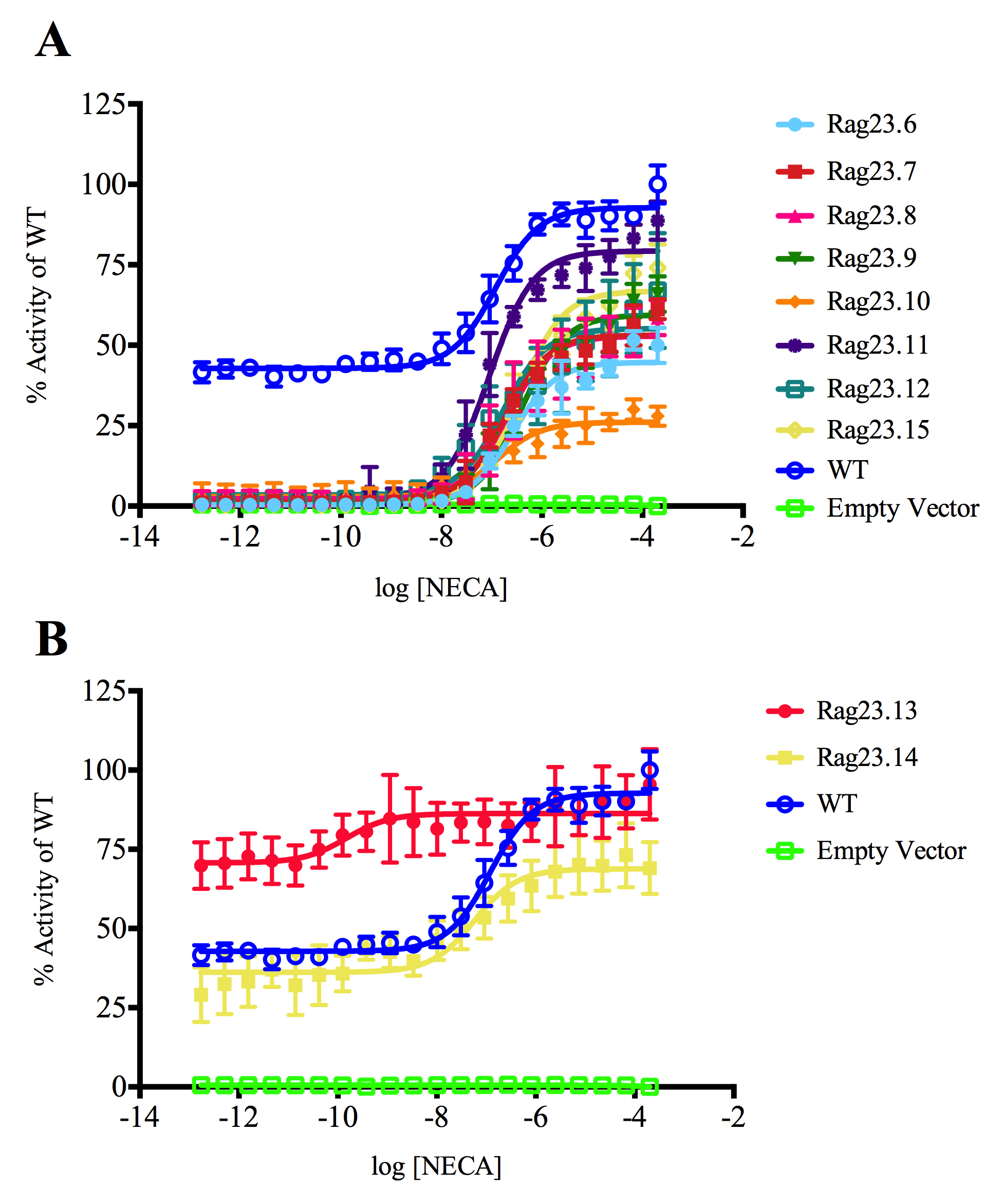
**

**Figure S2A and B** NECA-induced activity of the WT A_2A_R and the triple mutants intermediate between the WT and Rag23. See Table 1 for the precise details of each mutant. The receptor constructs were expressed in the MMY24 *S. cerevisiae* strain using the p306GPD vector. The activity of cells containing empty vector is shown as a control.

**
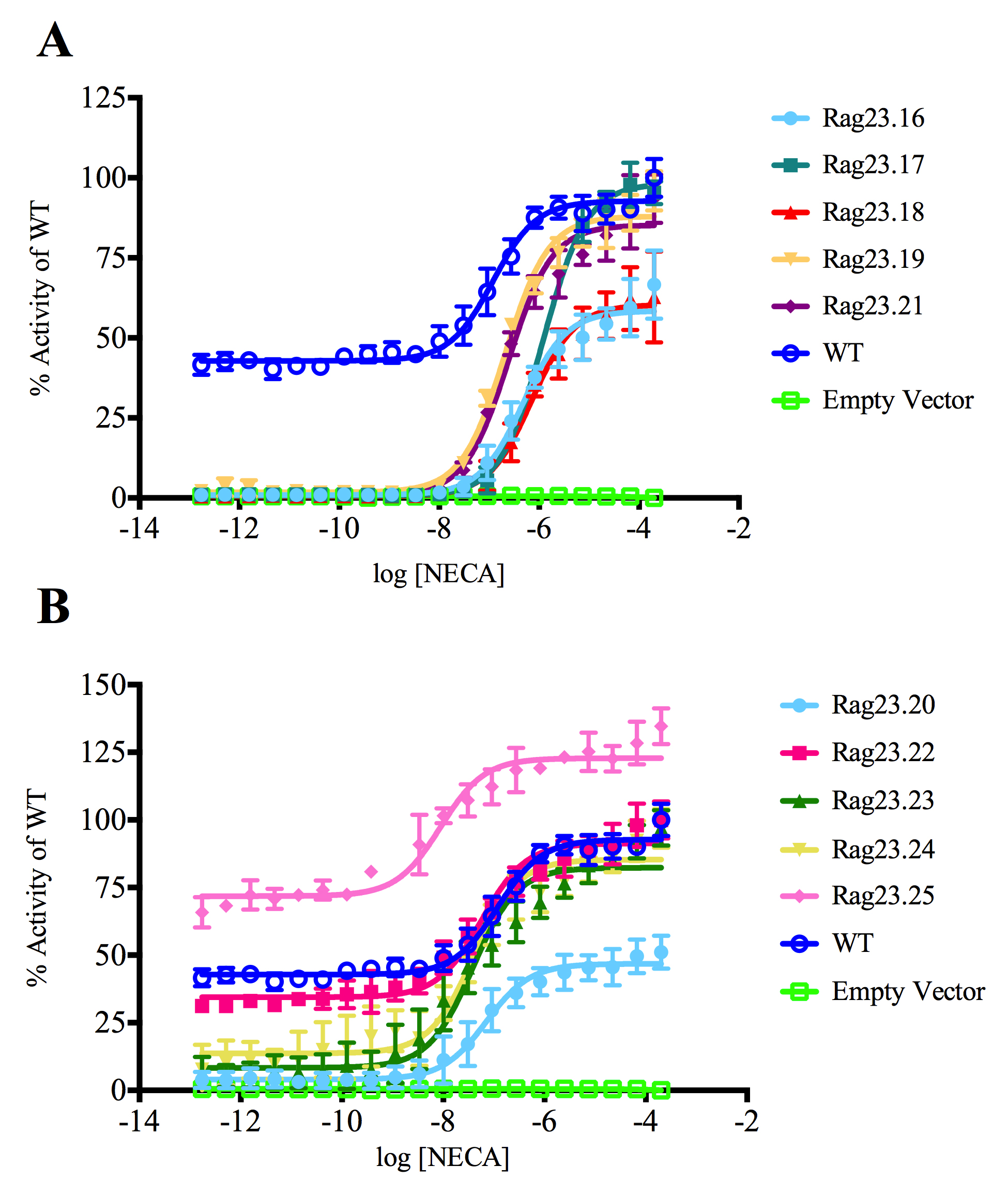
**

**Figure S3A and B** NECA-induced activity of the WT A_2A_R and the double mutants intermediate between the WT and Rag23. See Table 1 for the precise details of each mutant. The receptor constructs were expressed in the MMY24 *S. cerevisiae* strain using the p306GPD vector. The activity of cells containing empty vector is shown as a control.


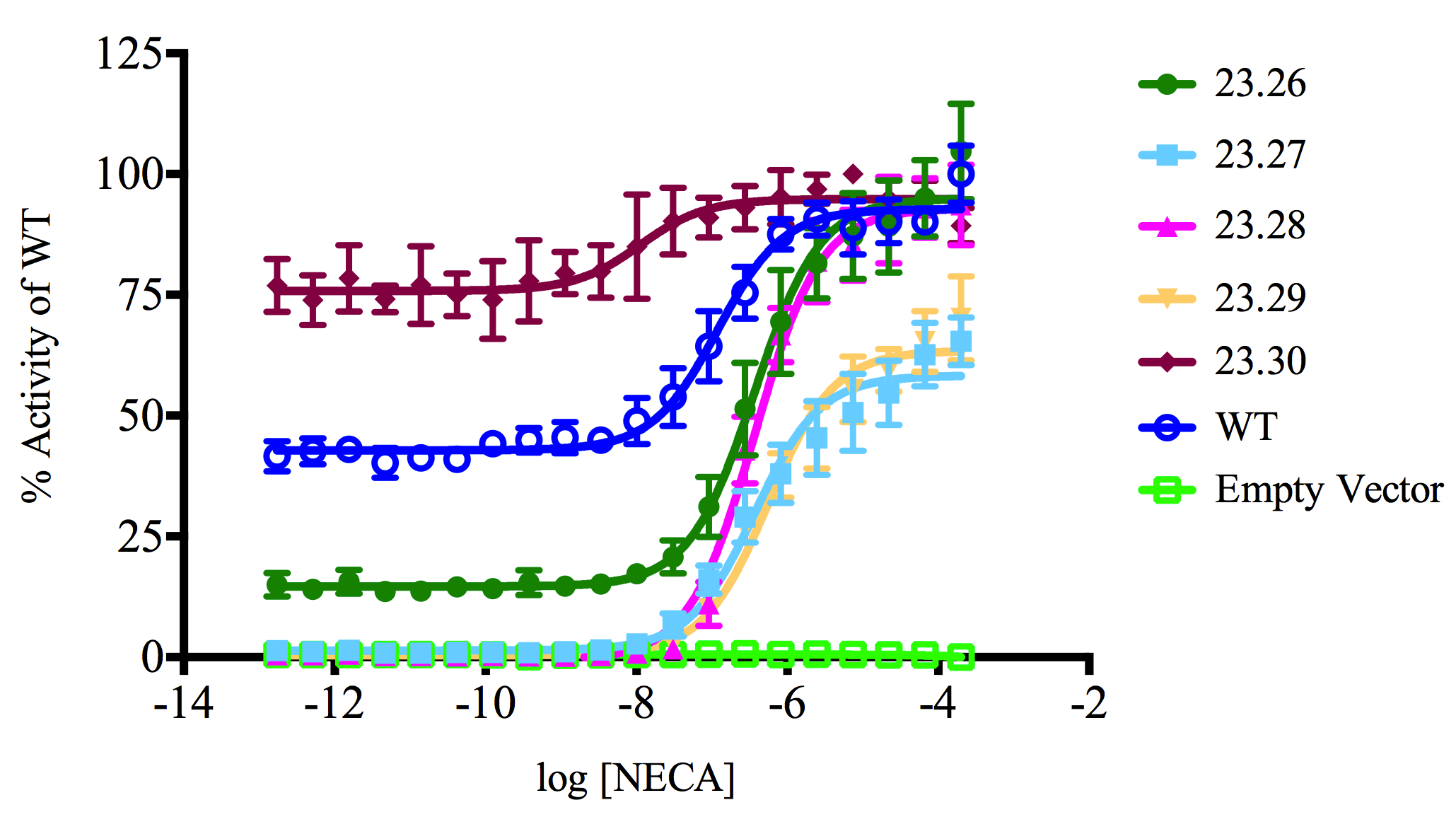


**Figure S4** NECA-induced activity of the WT A_2A_R and the single mutants intermediate between the WT and Rag23. See Table 1 for the precise details of each mutant. The receptor constructs were expressed in the MMY24 *S. cerevisiae* strain using the p306GPD vector. The activity of cells containing empty vector is shown as a control.
